# Differential Influence of the Dorsal Premotor and Primary Somatosensory Cortex on Corticospinal Excitability during Kinesthetic and Visual Motor Imagery: A Low-Frequency Repetitive Transcranial Magnetic Stimulation Study

**DOI:** 10.3390/brainsci11091196

**Published:** 2021-09-10

**Authors:** Viola Oldrati, Alessandra Finisguerra, Alessio Avenanti, Salvatore Maria Aglioti, Cosimo Urgesi

**Affiliations:** 1 Scientific Institute, IRCCS E. Medea, Neuro-Oncological and Neuropsychological Rehabilitation Unit, Bosisio Parini, 23842 Lecco, Italy; viola.oldrati@lanostrafamiglia.it; 2 Scientific Institute, IRCCS E. Medea, Pasian di Prato, 33037 Udine, Italy; alessandra.finisguerra@lanostrafamiglia.it; 3 Dipartimento di Psicologia, Centro Studi e Ricerche in Neuroscienze Cognitive, Alma Mater Studiorum-Università di Bologna, 47521 Cesena, Italy; alessio.avenanti@unibo.it; 4 Centro de Investigación en Neuropsicología y Neurociencias Cognitivas, Universidad Católica del Maule, Talca 3460000, Chile; 5 Sapienza, Università di Roma and CLN2S@Sapienza, Istituto Italiano di Tecnologia, 00161 Rome, Italy; salvatoremaria.aglioti@uniroma1.it; 6 IRCCS Fondazione Santa Lucia, 00179 Rome, Italy; 7 Scientific Institute, IRCCS E. Medea, Neuropsychiatry and Neurorehabilitation Unit, Bosisio Parini, 23842 Lecco, Italy; 8 Laboratory of Cognitive Neuroscience, Department of Languages and Literatures, Communication, Education and Society, University of Udine, 33100 Udine, Italy

**Keywords:** motor imagery, kinesthetic, visual, cortico-spinal excitability, transcranial magnetic stimulation

## Abstract

Consistent evidence suggests that motor imagery involves the activation of several sensorimotor areas also involved during action execution, including the dorsal premotor cortex (dPMC) and the primary somatosensory cortex (S1). However, it is still unclear whether their involvement is specific for either kinesthetic or visual imagery or whether they contribute to motor activation for both modalities. Although sensorial experience during motor imagery is often multimodal, identifying the modality exerting greater facilitation of the motor system may allow optimizing the functional outcomes of rehabilitation interventions. In a sample of healthy adults, we combined 1 Hz repetitive transcranial magnetic stimulation (rTMS) to suppress neural activity of the dPMC, S1, and primary motor cortex (M1) with single-pulse TMS over M1 for measuring cortico-spinal excitability (CSE) during kinesthetic and visual motor imagery of finger movements as compared to static imagery conditions. We found that rTMS over both dPMC and S1, but not over M1, modulates the muscle-specific facilitation of CSE during kinesthetic but not during visual motor imagery. Furthermore, dPMC rTMS suppressed the facilitation of CSE, whereas S1 rTMS boosted it. The results highlight the differential pattern of cortico-cortical connectivity within the sensorimotor system during the mental simulation of the kinesthetic and visual consequences of actions.

## 1. Introduction

Motor imagery (MI) refers to the mental simulation and subjective experience of movement in the absence of overt execution of the corresponding motor output [1]. It is a common notion that MI is underpinned by the motor representations in the brain largely overlapping with those involved in actual motor execution [2,3]. This notion has been reinforced by numerous neuroimaging studies that have examined the neural correlates of the mental simulation of action and have pointed to the activation of a broad network of cortical and subcortical regions known to be also involved in action execution (see [4] for a review). These include not only frontal motor areas, such as the premotor cortex (PMC) [5] known to be involved in action selection, planning, and preparation [6,7], but also parietal sensorimotor areas, such as the primary somatosensory cortex (S1) [8,9], which likely reflects the kinesthetic aspects of motor representations. Indeed, parietal sensorimotor areas have been hypothesized to generate sensory efference during MI even in the absence of movement execution [10]. An intriguing speculation proposes that regions hosting MI mechanisms are part of a complex circuit for the anticipation and prediction of the sensory consequences of movement, with the goal to optimize motor performance, depending on context demands [11]. Accordingly, following a forward-modeling perspective, mental motor representations and their resulting quasi-perceptual experiences of movement would derive from an internal prediction of the motor status of the corresponding movement based on a sensory efference copy [10,12,13].

In the light of the overlap between regions recruited during MI and actual movement execution [14], increasing attention has been devoted to explore the application of MI tasks in rehabilitation settings for patients suffering from a significant decrease in functional mobility [15,16], as well as in training for professional athletes [17,18] and musicians [19]. In these applications, it has become evident that MI can be performed by distinct modalities, with the most common two engaging kinesthetic and visual sensory experiences [20]. Kinesthetic MI (kMI) is a form of mental motor rehearsal focusing on how a movement “feels” in terms of perceptions deriving from our own body during the execution of the movement. Experimental instructions targeting kMI require participants to pay attention to the somatosensations that they would normally perceive during the execution of a movement, such as muscle stretching and contractions or tactile sensations. Visual motor imagery (vMI), conversely, mainly involves the visualization of a movement that can be either achieved from a first-person perspective, also referred to as internal vMI (i.e., with the image viewed by the subject’s own eyes), or from a third-person perspective, also referred to as external vMI (i.e., with the image viewed by an external observer’s standpoint).

Even if sensorial experience during MI is often multimodal [21,22], understanding which type of sensorial modality exerts greater facilitation of the motor system may allow better focusing of MI instructions in rehabilitation or training settings in order to optimize functional outcomes. Although there is consistent evidence suggesting that kMI and vMI may activate several common brain areas, modality-specific areas have also been identified. In particular, since the pioneering neuroimaging studies comparing kMI and vMI [23,24], kMI is associated with greater involvement of anterior motor areas, while vMI more consistently recruits posterior sensory areas. Recent studies, however, have reported mixed results [25,26,27,28,29]. These studies have shown PMC activation either during both kMI and vMI [25,26] or only during vMI but not kMI [27], whereas meta-analytic evidence shows a substantial overlap between the two modalities but more extensive recruitment of fronto-parietal sensorimotor circuits in the case of kMI [28]. This picture is further complicated by evidence suggesting that connectivity could characterize the functional role of brain activations to distinct imagery modalities better than local activity [30]. Brain co-activations across tasks, indeed, may reflect different excitatory or inhibitory influences on common areas. For example, it has been shown that while the pattern of parietal-motor functional connectivity is similar during movement execution and MI, it shifts from a facilitatory effect during movement execution to a suppressive effect during MI, arguably reflecting the suppression of motor output during MI [24] (see also [31] for a similar account for SMA–M1 connectivity during MI). In this sense, while premotor and sensorimotor areas may be activated during both kMI and vMI, their relative facilitatory and suppressive contribution to motor activation may vary in the two MI modalities.

While both excitatory and inhibitory activities are similarly represented in neuroimaging signals [32], a valuable assessment of motor activation during MI is provided by single-pulse transcranial magnetic stimulation (spTMS) experiments examining changes in cortico-spinal excitability (CSE). This experimental paradigm consists in targeting the primary motor cortex (M1) with a single TMS pulse and assessing the induced motor-evoked potentials (MEPs) recorded in specific muscles during MI. MEP amplitudes provide a fine-tuned measure of the excitatory/inhibitory influences on CSE during MI [33,34]. Indeed, increased MEP amplitude has been observed during MI compared to the rest condition for the muscle involved in the imagined movement, reflecting effective modulation of CSE by mental simulation of actions [33,35,36]. Remarkably, a previous spTMS MEP study found that kMI but not vMI of simple thumb abduction movements leads to CSE facilitation [37]. Conversely, in another study, CSE facilitation during kMI of simple finger movements (but not during vMI of the same movements) was affected by the congruency of the imagined and participant’s body posture [22]. Conversely, a later spTMS MEP study detected no difference in CSE between kMI and vMI when participants were required to mentally perform a complex finger-tapping sequence task [38]. In this study, however, participants reported difficulties in visualizing the movement sequence, which led them to change from vMI to kMI, as suggested by overall low vMI capabilities assessed through a self-report questionnaire. Thus, the relative contribution of MI to CSE facilitation may rely on different patterns of cortico-cortical connectivity, depending on both task characteristics and individual abilities in engaging in different imagery modalities [39,40].

A fundamental and yet entirely unexplored question is whether PMC and S1 functionally contribute to CSE facilitation during kMI and vMI. To address this outstanding question, here, we used a perturb-and-measure TMS protocol [41,42] with a group of 30 healthy humans. That is, we combined spTMS MEPs to assess CSE during kMI and vMI of simple finger movements (as in [37]), with low-frequency (1 Hz) repetitive TMS (rTMS), to suppress neural activity over the PMC—specifically over the dorsal PMC (dPMC), and the S1 and, thus, test their critical contribution to MI-related CSE modulation. Low-frequency rTMS over dorsal premotor cortex and M1 hand areas has been demonstrated to transiently suppress MEPs recorded from hand muscles for several minutes following stimulation [41,43,44,45] and can affect motor performance [45,46]. Few studies in which low-frequency rTMS was delivered over S1 showed impaired accuracy in tactile and proprioceptive tasks [47,48,49] and disrupted motor reactivity to observed overstretching hand movements [41], whereas it did not affect corticospinal excitability at rest [44,50]. These prior findings support protocol efficacy and selectivity in modulating motor and somatosensory processes. However, it is important to mention that none of these prior studies has explored rTMS-induced changes of CSE during MI. Since both dPMC rTMS [51] and S1 rTMS [52] have been reported to modulate CSE independently from MI, we also applied rTMS over an active control site, namely M1, that should similarly modulate CSE without affecting action simulation processes [41,53]. This way, we aimed to disentangle the specific causal contribution of dPMC and S1 to kMI- and vMI-related activations from the indirect (i.e., via M1) effects of dPMC and S1 rTMS on CSE.

Based on prior work [37], we expected larger and/or more consistent CSE facilitation during kMI as compared to during vMI of simple finger movements. Remarkably, we expected modality-specific effects following dPMC and S1 rTMS. In keeping with meta-analytic accounts that kMI might elicit more extensive clusters of activation in several fronto-parietal areas [28], we expected that rTMS over both dPMC and S1 would modulate CSE facilitation during kMI to a greater extent than during vMI. Given that rTMS over each node of the fronto-parietal network may either suppress or enhance CSE facilitation during MI according to the pattern of task-dependent excitatory or inhibitory influences on M1 [24], rTMS-related suppression of CSE facilitation during MI would point to an excitatory influence of the target area on M1, while rTMS-related enhancement of CSE facilitation during MI would point to an inhibitory influence.

## 2. Materials and Methods

### 2.1. Sample

Thirty healthy adult volunteers (15 female, M = 22.7, SD = 6.0 years) participated in this study and were randomly allocated to three groups receiving rTMS over the left M1, dPMC, or S1 for a total of ten participants per group. Both gender and age were homogeneously distributed across groups (gender: χ^2^(2,30) = 0.8, *p* = 0.67; age: F(2,27) = 0.91, *p* = 0.4). All participants were right handed and had normal or corrected-to-normal vision. Prior to the experiment, the participants filled in a questionnaire to evaluate their suitability for TMS. None of the volunteers had a history of neurological disorders, brain trauma, or a family history of epilepsy. Written informed consent was obtained from all participants before the beginning of the experiment. The study was approved by the ethical committee of the IRCCS E. Medea and conducted in accordance with the Declaration of Helsinki.

### 2.2. EMG and Single-Pulse TMS

EMG signals were recorded by means of a Viking IV electromyograph (Nicolet Biomedical Inc.) with 1-cm-diameter Ag-AgCl surface electrodes positioned, in a belly-tendon montage, over the first dorsal interosseous (FDI) muscle and over the adductor of the little finger muscle-abductor digiti minimum (ADM) of the right hand. The signal was amplified, bandpass-filtered online (20 Hz–3 kHz), and digitalized (sampling rate: 20 kHz). Auditory feedback of the electromyographic signal helped the participants to maintain voluntary muscle relaxation during the preliminary session. To make sure that there was no unwanted background EMG activity before the magnetic pulse, we had the signal of each muscle displayed additionally in separate channels set at high sensitivity (50 μV), which was continuously monitored by the experimenter during the experimental session.

TMS was performed with a Magstim 200 stimulator (Magstim Co., Ltd., Whitland, UK) connected to a 70 mm butterfly coil. At the beginning of the experimental session, spTMS was administered with the coil positioned tangentially to the scalp and over the left primary motor cortex (M1) to determine the individual resting motor threshold (rMT). The optimal scalp position (OSP) was set where larger and more stable MEPs were obtained (hotspot) from both muscles. The participants wore a tightly fitting bathing cap on which the scalp position for stimulation was marked. The coil was held by hand, and its position with respect to the mark was checked continuously to easily compensate for small movements of the participant’s head during data collection. The rMT was defined as the lowest stimulus intensity sufficient to evoke at least 5 MEPs out of 10 with an amplitude of at least 50 mV [54]. Because we aimed to evaluate the differential modulation of MEPs recorded from the FDI muscle during different imagery modalities, the rMT was determined for the FDI. In keeping with previous studies (see, e.g., [22,36,42,55,56]), the participants were stimulated at 120% of their rMT. With this intensity, we could obtain stable MEPs from both muscles in all conditions. The rMT did not differ among groups (F(2,27) = 0.09, *p* = 0.91, η_p_^2^ = 0.0; M1: M = 58.3, SD = 9.9; dPMC: M = 60.1, SD = 10.3; S1: M = 58.9, SD = 8.3). The MEP peak-to-peak amplitude (in microvolts) was collected and stored on a computer for offline analysis.

### 2.3. Procedure

The participants comfortably sat in an armchair with the right upper arm resting on a cushion (extended elbow, pronated forearm, and open, palm-down hand). At the beginning of the experimental session, the experimenter explained the task. The participants were asked to imagine performing abduction/adduction movements of their right index finger, without actually moving it, in four experimental conditions according to (i) a kinesthetic modality (condition 1: “Imagine feeling your hand moving, paying attention to the physical sensations that you may perceive from it.”); (ii) a visual modality (condition 2: “Imagine seeing your hand moving, in the clearest and most vivid way as possible.”); (iii) kinesthetic control (condition 3: “Imagine feeling your hand being still.”); and (iv) visual control (condition 4: “Imagine seeing your hand being still.”). Thus, the comparison between conditions 1 (dynamic) and 3 (static) was expected to probe the kinesthetic component of motor imagery, whereas the comparison between conditions 2 (dynamic) and 4 (static) was expected to probe its visual component. The participants performed the task with their eyes closed. The different task instructions were provided in separate blocks, and their order was counterbalanced across participants according to the Latin square procedure.

At the beginning of each trial, a short acoustic signal (beep) indicated to the participants that they had to start imagining their finger movement according to the instructions provided at the beginning of the block. After 5 s, another acoustic signal informed them to interrupt the imagery task. MEPs induced by spTMS delivered over M1 were recorded after a variable interval of 3–4.5 s from the beginning of the imaginative task in order to avoid any priming effects that could affect MEP size. The appropriate execution of the task was monitored by asking the participants to report where they imagined their index finger to be positioned at the moment of the TMS pulse delivery (i.e., abducted or adducted). An inter-trial interval of 2 s was set so that the interval between consecutive spTMS was 7 s on average.

MEP recording during the imagery task was carried out in two sessions. According to the perturb-and-measure approach, a session of spTMS coupled with MEP registration was performed immediately at the end of a 1 Hz rTMS train of 15 min (i.e., 900 pulses) delivered over the stimulation site defined by group assignment (i.e., left M1, left dPMC, or left S1). The choice of stimulating the left hemisphere was supported by evidence of larger MEP amplitudes in the right FDI (i.e., after left M1 spTMS) than in the left FDI (i.e., after right M1 spTMS) during MI of index finger flexions of the corresponding hand in right-handed individuals [57]. Indeed, during MI of hand actions, activation of cortical areas involved in motor planning and execution was found to be left-lateralized in right-handers [58].

In this “in-win-session” we recorded MEPs within the temporal window of rTMS inhibitory influence. In another, “out-win-session”, MEPs were recorded either before or at least 75 min after the end of rTMS, thus being well outside this window of rTMS influence (i.e., baseline). The order of these two sessions was counterbalanced between participants within each group. The choice of a 75 min interval between the in-win and out-win sessions aimed to minimize carry-over effects of rTMS, based on a prior study using the same perturb-and-measure paradigm during action observation [41]. The M1 site corresponded to the OSP. The dPMC site was localized 2 cm anteriorly and 1 cm medially to the OSP, following a previously applied procedure [45]. The S1 site was identified by moving the coil 1 cm posteriorly to the OSP on a line parallel to the interhemispheric scissure, in keeping with previous TMS studies that successfully targeted S1 with reference to the motor hotspot [59,60]. In all groups, the participants were stimulated at 90% of their MT during rTMS. The 1 Hz rTMS delivered over the left premotor cortex at 90% intensity was found to suppress the amplitude of MEPs in the right FDI, with the effect outlasting the full train of 1500 stimuli, thus, for at least 15 min [44].

**Figure 1:**
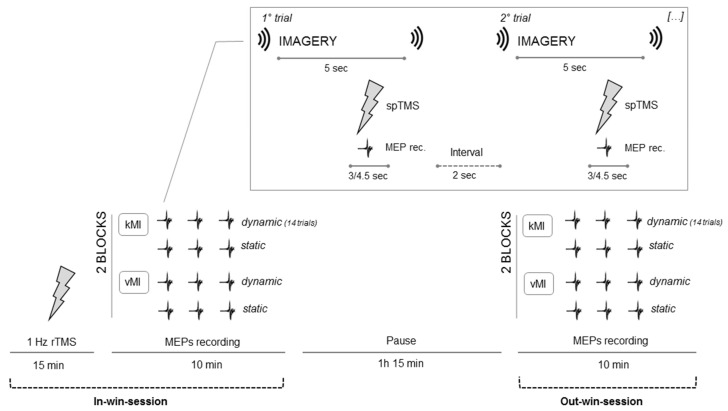
Schematic depiction of the experimental procedure. Participants performed the kinesthetic (kMI) and visual motor imagery (vMI) tasks coupled with the recording of MEPs right after a cycle of rTMS (in-win session) and before or at least 1 h and 15 min after the cessation of the stimulation (out-win session). Participants underwent either M1, dPMC, or S1 1 Hz rTMS according to group assignment. The box in the upper part depicts the MEP-recording trials, marked by acoustic signals, as carried out in each experimental block.

We recorded 14 MEPs in each of the four blocks, each one representing one of the four distinct experimental conditions, for a total of 56 MEPs per session. Thus, 112 MEPs were recorded per participant during the imagery task in each session, summing up to 3360 observations recorded across participants. Additionally, 7 MEPs were recorded for each participant at rest before and after the experimental procedure in both sessions, for a total of 14 MEPs serving as the resting-state condition of each participant per session. The total duration of each experimental block was approximately 1.5 min for an overall duration of each MEP-recording session, including coil positioning and a resting-state condition, of approximately 10 min, thus being within the time of 15 min, 1 Hz rTMS effects [41,45]. Figure 1 provides a schematic depiction of the experimental procedure.

In addition, at the end of the experimental procedure, the participants filled out the Italian version of the Revised Motor Imagery Questionnaire (MIQ-r) [36,55,61] to assess the subjective vividness experienced in generating kinesthetic and visual mental images. The MIQ-r requires the generation of four kinesthetic and four visual images of gross, whole-body movements (e.g., jumping) and the rating of the difficulty experienced during imagery with a 7-point Likert-type scale, with the descriptor extremes “very easy” (score 1) and “very difficult” (score 7). Therefore, lower scores indicate more successful imagery. Responses are summed per scale, thereby resulting in two scores, one for the visual and one for the kinesthetic imagery ability. Subscale scores can range from a minimum of 4 to a maximum of 28. The final scores were used as a proxy for individual imagery ability.

### 2.4. Data Handling and Statistical Analysis

First, a 3 × 2 ANOVA with the stimulation site (M1 vs. dPMC vs. S1) and imagery modality (kinesthetic vs. visual) as between- and within-subject variables, respectively, was conducted on the kinesthetic and visual MIQ-r scores to verify that both imagery abilities were balanced across groups.

For the main analysis, MEP amplitudes recorded from the FDI and ADM muscles were separately normalized per participant and per session, dividing each observation by the average of the MEPs recorded in the resting-state condition and multiplying the result by 100. Second, the normalized MEP amplitudes of the two muscles were combined together following the formula (FDI value/ADM value) × 100. This MEP ratio is considered to reflect the muscle-specific mapping of observed/imagined movements into the motor cortex [62,63]. In the present experiment, this index was used as a proxy of the effectiveness of the muscle specificity mapping of the task requiring imagining movements of the index finger. Values greater than 100 indicated that the FDI was more strongly involved than ADM in the imaginative task, as expected given the previous evidence of greater FDI than ADM activity during the observation of index finger abduction movements [55,64]. The obtained ratio was later used in the main analysis as the dependent variable.

A first linear mixed model (LMM) was estimated for the FDI/ADM ratio with SITE (left M1 vs. left dPMC vs. left S1), WINDOW (out-win session vs. in-win session), MODALITY (kinesthetic vs. visual), MOVEMENT (dynamic vs. static), and their 4-way interaction as fixed factors and intercepts and SUBJECTS as random factors. Supported by the results of this overall model, we tested our main hypotheses, namely modality specific facilitation of CSE during dynamic vs. static conditions in the out-win session and differential rTMS modulation of CSE in the in- vs. out-win sessions, in two separate follow-up analyses. To test whether the imagery task modulated CSE and whether CSE would be differently modulated by the imagery modality, an LMM was computed on the FDI/ADM ratio in the out-win session, serving as the baseline condition, with MODALITY and MOVEMENT and their 2-way interaction as fixed factors and intercepts and SUBJECTS as random factors. Finally, to explore the contribution of M1, dPMC, and S1 to the modulation of CSE passing from the out- to the in-win session, we calculated for each trial of the in-win session the difference between the FDI/ADM ratio in the in-win session and its mean in the out-win session for the same experimental condition. A positive value of this index denoted a facilitation of CSE, while negative scores indicated suppression. These difference values were entered into an LMM with SITE, MODALITY, and MOVEMENT and their 3-way interaction as fixed factors and intercepts and SUBJECTS as random factors. An advantage of LMMs is that they allow to account for random differences in outcome values between participants and/or between items or conditions characteristics [65]. Statistical significance was obtained by a type II Wald chi-square. Post hoc comparisons were carried out applying Tukey’s adjustment. All data from the LMMs are reported as estimated marginal means ± standard error of the mean (SEM). All the models were estimated using R (version 4.0.3) and the lme4 R package (version 1.1.23) [66].

## 3. Results

The 3 (stimulation site) × 2 (imagery modality) ANOVA on kinesthetic and visual MIQ-r scores revealed that the main effect of groups (F(2,27) = 0.2, *p* = 0.8, η_p_^2^ = 0.01) and its interaction with imagery modality (F(1,27) = 0.04, *p* = 0.96, η_p_^2^ = 0.002) were not significant, indicating that the three groups displayed comparable kMI and vMI abilities. However, the analysis yielded a main effect of modality (F(2,27) = 8.4, *p* < 0.01, η_p_^2^ = 0.14). Indeed, the participants found it overall more difficult to generate kinesthetic (12.1 ± 0.71) than visual mental images (9.5 ± 0.52).

The main analysis aimed at exploring the variation in the MEP ratio amplitude according to the experimental conditions. The four-way LMM on the FDI/ADM ratio yielded a significant four-way interaction of SITE*WINDOW*MODALITY*MOVEMENT (χ^2^(18) = 84.14, *p* < 0.0001). This revealed that CSE facilitation during dynamic vs. static imagery is differently modulated by rTMS over the three target areas in the two modalities. The model did not yield any significant main effect (all *p* > 0.2). Table 1 reports mean FDI/ADM ratio values for each factor included in the model.

To explore the source of the significant four-way interaction, we first tested the hypothesis that kMI is associated with greater CSE facilitation in the dynamic vs. static condition as compared to vMI. The LMM on the FDI/ADM ratios in the out-win session, collapsing all stimulation sites together, yielded a significant main effect of MOVEMENT (χ^2^(1) = 4.28, *p* < 0.05) and a significant interaction effect of MODALITY*MOVEMENT (χ^2^(1) = 12.45, *p* < 0.001). Indeed, overall, greater FDI/ADM ratios were observed in the dynamic condition (129% ± 9.18%) than in the static condition (119% ± 9.20%). Post hoc analysis of the interaction effect, however, showed greater FDI/ADM ratios in the dynamic condition (138% ± 9.74%) than in the static condition (112% ± 9.75%, *p* < 0.001) during kMI, whereas such a difference did not emerge during vMI (*p* = 0.72). Moreover, post hoc analysis showed a greater MEP amplitude ratio in the kinesthetic dynamic (138% ± 9.74%) than in the visual dynamic condition (120% ± 9.76%, *p* < 0.05). The main effect of MODALITY was not significant (*p* = 0.6). This confirmed that kMI, but not vMI, leads to a significant facilitation of CSE at baseline (i.e., out-win session).

**Figure 2:**
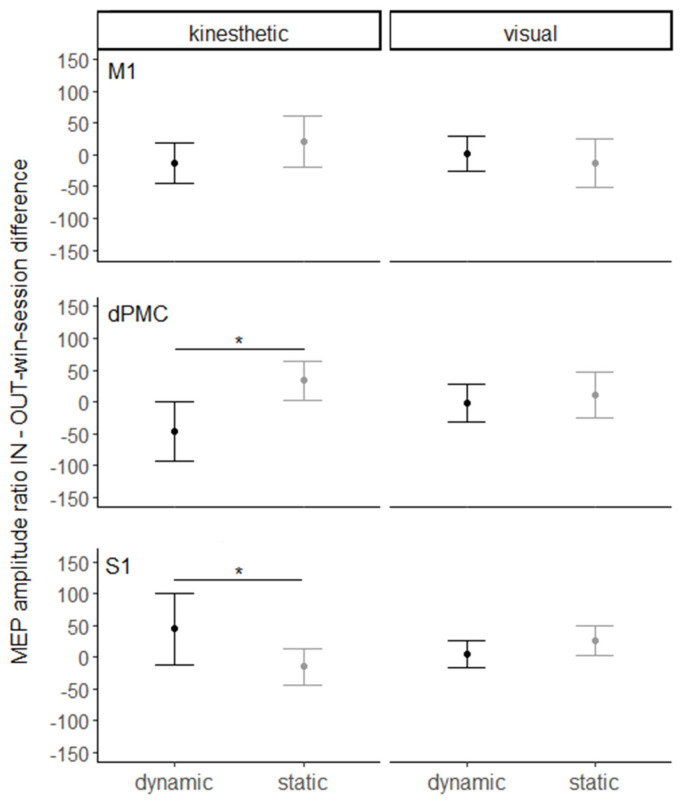
Estimated marginal mean ± standard error of the mean (SEM) of the FDI/ADM ratio difference between the in-win and out-win sessions as a function of the stimulation site (M1 vs. dPMC vs. S1), imagery modality (kinesthetic vs. visual), and movement (dynamic vs. static). Asterisks indicate significant differences (*p* < 0.01).

To test how this pattern of CSE facilitation is modulated by the rTMS of the three target areas, an LMM was computed on the difference between the FDI/ADM ratios in the in-win and out-win sessions, including the factors SITE, MODALITY, and MOVEMENT (Figure 2). The analysis yielded a significant main effect of MOVEMENT (χ^2^(1) = 4.13, *p* < 0.05), driven by a greater rTMS-related suppression of MEP amplitude ratio in the dynamic (−1.39 ± 12.6) as compared to the static condition (10.47 ± 12.5). The interaction effect of SITE*MODALITY*MOVEMENT was also significant (χ^2^(7) = 57.51, *p* < 0.0001). Post hoc analysis indicated a greater suppression of MEP amplitude ratios in the dynamic condition (−45.43 ± 22.9) than in the static condition (33.86 ± 22.8, *p* < 0.001), passing from the out- to the in-window of dPMC rTMS perturbation. Conversely, passing from the out- to the in-window of S1 rTMS perturbation, a greater facilitation of MEP amplitude ratios was observed in the dynamic condition (44.73 ± 22.8) as compared to the static condition (−15.07 ± 22.8, *p* < 0.01). All other effects were non-significant (other *p* > 0.8).

Lastly, to verify the absence of carry-over effects, an unpaired two-samples *t*-test on MEP ratios in the out-win sessions performed before or 75 min after the delivery of rTMS was computed. The analysis yielded a non-significant effect (t(26) = −0.5, *p* = 0.6).

## 4. Discussion

In the present study, we used a perturb-and-measure TMS paradigm [41,42,67] to explore the modulation of CSE during kMI and vMI. In the light of evidence of CSE facilitation following kMI but not vMI [37], we expected greater CSE facilitation during kMI than during vMI. Furthermore, considering meta-analytic accounts of broader clusters of activation in fronto-parietal areas during kMI as compared to vMI [28], we also expected rTMS over both dPMC and S1 to modulate CSE facilitation during kMI to a greater extent than during vMI. Finally, we aimed to explore the contribution of the dPMC and S1 to motor activation during the two imagery modalities. These brain regions have been observed to be consistently activated during MI [68]. However, it is still unclear whether the dPMC and S1 involvement is specific for either kMI or vMI or whether instead they contribute to motor activation for both modalities but with different patterns of excitatory/inhibitory influences. To address this issue, we combined 1 Hz repetitive TMS to suppress the neural activity of the left dPMC, S1, and M1 with single-pulse TMS to measure CSE during kMI and vMI of simple finger movements as compared to static imagery conditions. First, in line with previous evidence [37], the analysis on the baseline values registered outside the window of inhibition created by rTMS showed a facilitation of CSE during kMI but not during vMI. The analysis of the changes of CSE after rTMS further specified this finding, indicating that rTMS over both the dPMC and S1, but not over M1, modulates the muscle-specific facilitation of CSE. Furthermore, we observed a suppression of CSE in the dynamic kMI condition passing from the out- to the in-win session of perturbation created by dPMC rTMS. An opposite pattern of results emerged following the stimulation of S1, where an increase in CSE facilitation for the dynamic compared to the static kMI condition was observed passing from the out- to the in-window of rTMS inhibition. Thus, the muscle-specific facilitation of CSE for the dynamic as compared to the static kMI condition was suppressed by the inhibitory rTMS of the dPMC, but it was enhanced by the temporary rTMS inhibition of S1. The selectivity of the effects for dynamic kMI, their opposite direction after dPMC and S1 rTMS, and the absence of specific CSE changes after M1 rTMS rule out that the results could be due to indirect, non-specific effects of rTMS on CSE. Rather, the results hint at the differential pattern of cortico-cortical effective connectivity within the sensorimotor system during the mental simulation of the kinesthetic and visual consequences of actions.

The fact that the rTMS of both the dPMC and S1 affected only kMI, but not vMI, is in keeping with converging evidence that kIM shares more psychological and physiological aspects with movement execution as compared to vMI [23,24,33]. Indeed, it has been previously suggested that kMI may modulate cortico-excitability to a greater extent than vMI owing to the differences in the organization of the somatosensory-motor and visuo-motor systems, respectively, involved in the sensation of “feeling” or “seeing” ourselves performing a movement [37,56]. In a similar vein, kMI was shown to elicit more extensive clusters of activation in several fronto-parietal areas and, in particular, in anterior sensorimotor nodes [28]. Conversely, vMI more extensively modulated visual cortex areas [24]. Although both modalities were seen to be recruited, our participants reported to have better vMI than kMI abilities. This may suggest that the modality-specific facilitation of CSE for kMI observed in the present study is unlikely owed to the inability of participants to form visual mental images, but it more likely reflects the activation of a greater network including areas of the motor system during kMI as compared to vMI [28,37]. Interestingly, when considering neuroimaging studies exclusively recruiting athletes, no clear distinction was found to emerge between neural networks sub-serving kMI and vMI [40]. According to the authors of this meta-analysis, a possible explanation for this result is that athletes show greater integration between modalities than non-athletes—as were the participants included in the present study—since, thanks to their superior imagery abilities, sportspersons may tend to recruit sensorimotor areas also during vMI. The role of MI abilities is also particularly relevant in rehabilitation settings, where damage to different nodes of the premotor-parietal network may differently hinder the engagement of kinesthetic and visual sensorial modalities during MI. Thus, future studies are needed to better understand the effects of different types of fronto-parietal damage on the abilities to be engaged in the two imagery modalities in order to better tailor imagery-related rehabilitation interventions.

Our finding of decreased muscle-specific facilitation of CSE during kMI following rTMS over the dPMC falls in line with previous evidence of strong activity of this area during kMI [25,26,29,30]. The dPMC activation during kMI was linked to an increase in the MEP amplitude of hand muscles following spTMS over the relative OSP [69]. Similarly, Szameitat and colleagues [70] reported strong activation of the left dPMC during kMI of movements with the contralateral hand, endorsing the role of the dPMC in motor planning and control of motor mental images. In keeping with these findings, we showed that the transient suppression of dPMC cortical activity hinders the CSE facilitation elicited by the kinesthetic components of MI, suggesting that the dPMC may be involved in the motor planning mechanisms not only during motor execution but also during MI. The absence of effects for vMI is instead in contrast with findings showing dPMC involvement during vMI, which has been detected in several neuroimaging studies [26,27]. However, it should be considered that in our experiment, we failed to detect muscle-specific CSE facilitations during vMI, and therefore, whether the dPMC contributes to CSE modulations during vMI should be addressed in future studies using different tasks (e.g., more complex movements) or participants (e.g., athletes), leading to CSE facilitation also during vMI.

In a similar vein, that CSE facilitation during dynamic kMI is affected by S1 rTMS is in keeping with neuroimaging studies showing the involvement of S1 activation, in particular during kMI [25,29,30]. Remarkably, our findings support the notion that connectivity between S1 and M1 during MI may be inhibitory [24], since we found that transient suppression of S1 boosts the facilitation of CSE during kMI. Such inhibitory S1–M1 interaction would serve to prevent overt motor execution, while activating the kinesthetic sensorial consequences of the imagined movements, suggesting that in the healthy brain, motor execution is normally inhibited during imagery throughout interactions between sensorimotor regions [71]. Indeed, the parietal cortex has been proposed to contribute to MI by storing and/or gating the access to motor representations, as suggested by evidence of impaired ability to estimate hand motor performance through mental simulation in patients with parietal lobe lesions [72]. Furthermore, a single case study described a stroke patient who suffered from bilateral parietal damage and could not avoid to execute the actions he imagined [73]. In keeping with the notion that MI recruits forward models to predict and estimate the sensory consequences of imagined movements [10,73,74], the symptom of executing the imagined movements could be caused by a dysfunction of the forward model circuitry, with failure to inhibit the expected motor consequences embedded in the model itself. Accordingly, the rTMS disruption of somatosensory processing in S1 may have affected the processing of the sensory and motor in-/outflow of information, possibly enhancing anticipatory processing and determining the observed CSE facilitation in M1.

In this line, the increase in CSE facilitation after S1 perturbation may also hint at the involvement of compensatory mechanisms. This hypothesis goes in the same direction of the findings of a lesion study conducted by Johnson and colleagues [75]. In this study, chronic hemiplegic patients without premotor and parietal damage were more accurate in a series of MI tasks involving their contralesional than ipsilesional upper limb. Conversely, control patients who regained nearly all limb functionality following an initially severe hemiparesis did not display such difference. This finding not only may demonstrate that kMI is not abolished by the lack of sensory feedback but also that rather it may be facilitated by its removal. The authors suggested that this so-called “hemiplegic advantage” may be related to an enhanced motor planning effort of the affected limb. Case and colleagues [76] proposed an alternative explanation for this result, arguing that imagery might be strengthened in the case of reduced availability of sensorimotor feedback caused by the disruption of proprioceptive monitoring, as the that experienced by hemiplegic patients. The authors also pointed out that this explanation, alternative to that of the motor planning effort, is more in accordance with evidence of poor MI performance in healthy individuals with anesthetized arms [77]. Indeed, while hemiplegic patients have impaired proprioceptive processing of their limb position, healthy individuals with anesthetized arms likely maintain proprioceptive representations of their arms, which could conflict with the movements rehearsed during MI. Taken together, these findings suggest that interfering with proprioceptive processing may result in a facilitation of MI, providing a theoretical support for our findings of enhanced CSE facilitation during kMI after rTMS-induced perturbation of S1 and supporting the use of kMI to boost motor activation and recovery of motor functions after sensorimotor damage.

Neuroimaging meta-analyses suggest that M1 is not consistently activated during MI [28], whereas classical spTMS studies have shown that MEPs induced by M1 stimulation are typically enhanced during MI of manual movements [78,79]. These findings have been interpreted as evidence of higher sensitivity of spTMS MEPs than fMRI to subthreshold motor activation in M1. Alternatively, they could suggest that changes in CSE during MI reflect activity in brain regions interconnected with M1 (rather than M1 itself). Our perturb-and-measure approach supports this second hypothesis, as we found no effect on MI-related CSE facilitation following rTMS over M1. Our findings suggest that CSE facilitation during MI is likely driven by the activation of other nodes of the fronto-parietal network involved during MI, in particular premotor and somatosensory areas, which may facilitate CSE via either cortico-cortical or cortico-spinal connections [24]. Our result echoes the results obtained after using a perturb-and-measure approach to investigate the contribution of motor, premotor, and somatosensory areas to CSE facilitation during action observation [41]. Indeed, while rTMS of premotor and somatosensory cortex modulated motor facilitation during action observation, no specific effects beyond a general reduction in CSE were observed after M1-rTMS, suggesting that the muscle-specific CSE facilitation during action observation [64,80] is likely driven by connections of M1 with other fronto-parietal areas [41].

The conclusions that can be drawn from this study must be considered in the light of its limitations. To start, the sample size was modest. Besides the issue related to the inter-individual variability in TMS outcomes [81], such sample size may detract from the specificity of dPMC and S1 involvement in kMI but not in vMI. Indeed, it is possible that the effects for vMI were smaller and could not be detected with the power allowed by the study sample size. Then, due to the time constraints of rTMS-induced effects, we could not measure subjective reports of predominant use of a kinesthetic or visual strategy in each condition; hence we cannot be sure of the participants’ compliance with the instructions provided within each experimental block. Thus, the visual components may have been elicited by kMI and vice versa. Nevertheless, the different effects of the dPMC and S1 on kMI vs. vMI suggest that different processing strategies were predominantly triggered in the two conditions. Furthermore, the participants’ ability to generate kinesthetic and visual images was assessed with the MIQ-r and was considered as adequate. Furthermore, the lack of neuro-navigation in locating the stimulation targets must also be acknowledged as a limitation of the study. Another potential issue is represented by the time interval set between the out- and in-win sessions. Although a 75 min interval was enough to minimize carry-over effects in a prior perturb-and-measure study [41] and this interval is more than three times longer than the effect observed by Romero et al. [82] following subthreshold (90% intensity) 1 Hz rTMS targeting M1 for 10 min, the remaining carry-over effect cannot be excluded. Such a possibility has to be considered, given that rTMS has also been proven to induce cumulative plastic changes in motor cortex excitability after 24 h [83]. Nonetheless, varying the order of the two sessions across participants allowed us to minimize the influence of any carry-over effects on our results. Finally, since the recording of EMG activity was triggered by the TMS pulse, we could not control for subthreshold EMG pre-activations during MI, which have been found to be consistently elicited during kMI and may contribute to the pattern of CSE facilitation [34].

## 5. Conclusions

In conclusion, despite their limitations, the present findings provide evidence of a differential contribution of the dPMC and S1 to the processing of kinesthetic mental images of movements. They also provide further support to the exploration of kinesthetic-based MI protocols in the context of rehabilitation to facilitate motor recovery. Indeed, the stronger involvement of the dPMC and S1 in kMI than in vMI may explain the better outcomes attributed to rehabilitation programs for the improvement of motor functionality focusing on the kinesthetic more than the visual strategy [84,85].

## Figures and Tables

**Table 1:** Estimated marginal means ± standard error of the mean (SEM) of the FDI/ADM % ratio as a function of the rTMS session (out-win session vs. in-win- session), stimulation site (M1 vs. dPMC vs. S1), imagery modality (kinesthetic vs. visual), and movement (dynamic vs. static).

	**Out-Win Session**			
	**Kinesthetic MI**		**Visual MI**	
	Dynamic	Static	Dynamic	Static
M1	130.7 (14.0)	125.2 (14.2)	129.3 (14.2)	151.7 (14.5)
dPMC	158.4 (14.0)	98.3 (14.0)	132.1 (14.0)	137.3 (14.0)
S1	125.8 (14.1)	115.9 (14.0)	98.3 (14.1)	96.1 (14.0)
	**In-Win Session**			
	**Kinesthetic MI**		**Visual MI**	
	Dynamic	Static	Dynamic	Static
M1	120.3 (14.2)	145.5 (14.1)	130.9 (14.0)	129.7 (14.1)
dPMC	114.0 (14.1)	132.0 (14.1)	130.5 (14.1)	148.0 (14.0)
S1	170.0 (14.0)	100.7 (14.1)	103.5 (14.1)	123.5 (14.0)

## Data Availability

The datasets generated and analyzed during the current study are available in the Zenodo repository: doi:10.5281/zenodo.5126583.
